# Genomics and Pain Research in Sickle Cell Disease: An Explanation of Heterogeneity?

**DOI:** 10.5402/2011/672579

**Published:** 2011-04-20

**Authors:** Maxine Adegbola

**Affiliations:** College of Nursing, University of Texas at Arlington, 411 S. Nedderman Drive, Arlington, TX 76019, USA

## Abstract

Sickle cell disease (SCD) is a chronic illness, and the major complication, pain, results in complex multidimensional problems that affect an individual's ability to maintain adequate quality of life in multiple areas. Chronic SCD pain is inadequately treated, because it is not well understood, and the degree of chronic pain, clinical presentation, and sequela complications can vary from patient to patient, even among individuals with the same SCD genotype. The reason for this variation is unknown, but the underlying cause might be genetic. Researchers have not explored the contribution of a genomic variable to the occurrence of heterogeneous chronic SCD pain. Previous research on the *guanosine triphosphate cyclohydrolase* (GCH1) gene suggests that in some cases, phenotypic heterogeneity in human sensitivity to pain correlates with underlying genotypic variations in the GCH1 gene. These findings imply that genotypic variations might also explain why some SCD patients experience more chronic pain than others.

## 1. Introduction

The chronic pain in sickle cell disease (SCD) is a complex, distressing, and multidimensional problem that has no satisfactory therapy and needs more focused research. Patients suffering from SCD frequently complain of chronic, debilitating pain, yet, the degree of that pain varies from patient to patient, even among individuals with the same genotype. With patient-to-patient variation (heterogeneity) of pain, one might ponder: do genes influence how much chronic pain variability individuals with SCD experience? 

There is an evolving body of literature that provides evidence that chronic pain experiences in other diseases or conditions are associated with genetic influences of pain genes [1–6]. Even though SCD is a genetic disorder, the effect of genomics on the presence of chronic pain in SCD has not been examined. By incorporating molecular genetic pain findings into research on pain experiences in SCD, emerging information may help to explain the heterogeneity of chronic pain ratings among those with SCD and other chronic pain syndromes. With significant discoveries of how genes and allelic variations can modulate pain sensitivity and variation among individuals [1–6], it may become routine practice in the future to screen individuals with chronic pain for genetic susceptibility, and hence provide personalized care [7] and treatment relative to an individual's genetic predisposition.

The purpose of this paper is to introduce researchers and practitioners to current findings about a candidate gene's contribution to pain and to highlight the need to include a genomic variable when evaluating chronic pain in individuals with SCD. Since evidence shows that pain genetics play an important part in chronic pain trajectory [1–6], inclusion of genomic information could impact the identification of susceptibility to chronic pain, management strategies, care outcomes, and overall quality of life of individuals. A better understanding of the major complications of SCD, chronic pain, and the genomic effect of pain-protective genes may help to explain heterogeneity of chronic pain occurrences and why the experiences of pain vary from patient to patient.

## 2. Background

### 2.1. Sickle Cell Disease

SCD is a single-gene disorder resulting from point mutation and deoxyribonucleic acid (DNA) malfunction in which a single base pair in the nucleotide is affected. At the cellular level, on codon 6 of the hemoglobin gene, glutamic acid is replaced by valine because of the chromosomal defect. The result of this genetic mutation is protein change, multisystem defects, and pain [8, 9]. 

#### 2.1.1. Epidemiology

SCD affects over 70,000 to 100,000 individuals in the US [10, 11] and approximately 300,000 infants who are born annually throughout the world, with most of these infant births being in developing countries in Africa [12]. SCD mainly affects individuals of African, especially sub-Saharan, descent and occurs in approximately one out of every 500 births among African Americans and one out of 1,400 births among Hispanic Americans [13]. Hispanic Americans are part of the emerging SCD population. With easy and rapid global transcultural travel, and an admixture of individuals from varying ethnicities, SCD prevalence will increase.[12, 14, 15]. Hence, SCD affects many individuals worldwide who were not originally included in the disease susceptibility forecast and is a global public health concern [16]. Those affected by SCD are faced with multisystem, multidimensional problems that impinge on every domain of human life and include biophysical, sociological, psychosocial, and spiritual malfunctions.

### 2.2. Chronic Pain

Chronic pain is a disease unto itself [17] and a serious public health problem that is associated with increased risk of death [18]. With recognition that chronic pain is a disease consisting of a constellation of signs and symptoms, providers are called to diagnose and treat the condition and the intense accompanying complications that cause further health problems. Chronic pain results in persistent discomfort and inadequate care for individual sufferers. [19]. Accompanying chronic pain are sequela conditions that can lead to psychological changes such as sleep disturbances [20, 21] and psychopathology such as depression, anxiety and personality disorders [22, 23], brain damage, altered neurochemistry, and atrophy [17], which may be irreversible. Unrelieved pain affects social lives, results in pecuniary burdens [24] and social and psychological losses [24–27] and places economic burdens on society [28]. 

In general, the definition of chronic pain remains nebulous and temporal. The International Association for the Study of Pain [29] and American Pain Society [30] refer to chronic pain as pain occurring beyond normal tissue healing time, 3 months. Unlike acute pain, chronic pain serves no known adaptive purpose [31], may reflect ongoing tissue damage [28, 32, 33] including brain damage [17], and often leads to a burdensome life path [28]. 

Providers widely view chronic pain as a multifaceted, biopsychosocial, and spiritual phenomenon that can be associated with psychopathology [14] and which is best managed using a holistic, multidisciplinary approach [22]. Individuals with chronic pain should be treated early and aggressively to minimize altered brain physiology, poor memory, and other complications [17]. 

#### 2.2.1. Chronic Pain with SCD

Chronic pain, in general, is inadequately defined, and SCD-related chronic pain is poorly characterized and sparsely researched [33, 34]. Chronic aching pain, the hallmark and defining feature of SCD, occurs in addition to periodic acute pain. The chronic pain varies in intensity and duration and is considered to be unrelenting and crippling [35, 36].

Individuals with SCD are primarily of minority ethnicities. Their reports of pain are often underestimated and undertreated [30], and they are at risk for inadequate treatment within existing healthcare systems [37, 38]. The chronic pain is often managed on an outpatient basis with analgesics and adjuvant therapy, but both patients and healthcare providers express concerns about inadequate pain management [37, 38]. Moreover, clinicians and researchers do not agree on chronic pain definition, classification, or appropriate management strategies that actually meet the needs of individuals with SCD.

Chronic pain, the major presenting symptom of SCD, is complex, poorly understood, and the leading reason for hospitalizations and emergency department visits [39]. The pain is unrelenting, unpredictable, and different for each individual [40]. Daily experiences differ from prior experiences and become the focus throughout the individual's life, thus negatively affecting one's quality of life (QOL) [41, 42]. Recent research suggests that patients with SCD have more pain than was previously reported, have constant pain most days, self-manage the pain at home, and report increased pain despite opioids for analgesia. The pain often intensifies and necessitates healthcare utilization such as emergency room visits [39, 42]. The pain, at times, reflects a combination of chronic pain as a basal event with superimposed escalations of acute pain. Hence, the pain may be similar in type, location, or quality to usual chronic pain, but the severity is increased [43]. Individuals with SCD report experiential knowledge of being able to distinguish the pain type and can differentiate pain related to chronic everyday SCD pain from nonsickle cell disease-related pain and from chronic pain with increased acute intensity or exacerbation [44]. 

Published guidelines for the *Management for Sickle Cell Disease* identify chronic pain as a unique syndrome that occurs after approximately 3-to-6 months [30] after injury. In this guide, however, there is no offered explanation for the heterogeneity (patient-to-patient variation) of chronic pain experience that occurs even among individuals with the same sickle cell genotype. For individuals with SCD, the generic definition of chronic pain that has been used is the same for other disease processes and events and is unclear. The temporal presentation and nomenclature of chronic pain in individuals with SCD does not aptly fit this generic chronic pain description and possibly needs to be revisited and in the future clarified. Varying presentations such as frequency, intensity, and duration of chronic pain of SCD genotype need to be clarified and defined from the perspective of an individual with the illness. This, combined with genetic studies, might reveal some clues about genes that influence SCD chronic pain. 

### 2.3. Genomics and Pain

Even though the pain phenotype represents a subjective perception and is often difficult to measure, there is a need for more quantitative data on chronic pain phenotypes and genomic predisposition. The molecular epidemiologic mechanism of pain can better explain the phenotype. Since pain is no longer attributed solely to neurophysiologic changes, the study of pain must include genomic inquiry [45], which should include genetic contribution to pain itself, pain as a disease, and genetic factors playing a role in variability of pain experiences. Evolving information on pain protective genes has implicated genetic predisposition to chronic pain. With such emerging discoveries on pain genetics, clinical practice might have better or more poignant tools to help direct and predict care of those with chronic pain. This burgeoning body of pain genetic research suggests that variability in the gene loci contributes to individual experiences in pain sensitivity [46, 47]. Even in multifactorial pain syndromes, there is substantial heritability [1–6]. Two major approaches to conducting pain genetics research are genome wide association studies (GWAS) and cadidte gene studies where specific mutations (polymorphisms or single nucleotide polymorphisms [SNPs]) in candidate genes have already been identified. The candidate gene approach is hypothesis driven and targeted to specific pre-examined SNPs. Conversely GWAS are not hypothesis driven and tend to be untargeted and spread across the whole genome [48].

Pain genetics research helps to explain chronic pain mechanisms and genomic variability in individuals' susceptibility to chronic pain disorders. Genetic variables associated with chronic pain may help to clarify pathophysiologic mechanisms and help to identify patients who are at risk for developing chronic pain and other complications [49]. The long-term promissory outcome includes personalization of pain treatment in response to individual genomic structure. 

#### 2.3.1. A Pain Protective Gene: GCH1

In order to understand the potential value of examining the relationship of genetics with SCD, examining the research related to *guanosine triphosphate cyclohydrolase* (GCHI) gene can provide some beginning insight. Emerging genetic research has identified single nucleotide polymorphism (SNP) variations (mutations) in pain protective genes that contribute to protection or minimization against chronic pain [50–53] and individual differences in pain sensitivity. These relationships have not been examined in African Americans (AA) or in those with SCD and may contribute to understanding of this complex condition.

Recent studies suggest that polymorphisms in the *guanosine triphosphate cyclohydrolase* (GTP cyclohydrolase) (GCH1) gene within chromosome 14Q22-Q22.2 provide pain protection [52, 53]. SNPs in GCH1 (rs8007267, rs3783641, and rs10483639) have been associated with reduced tetrahudrobiopterin (BH4) levels and reduced pain sensitivity among adults with chronic pain. [50, 54] Individuals with GCH1 gene variant (expressed uncommon alleles) have less chronic pain.

The GCH1 pain protective haplotype has been associated with lower pain ratings in healthy volunteers in four independent studies [50–52, 55] and in individuals with chronic pain in two studies [52, 55]. In the Tegeder et al. [52] study, the researchers reported that specific genetic variations in the GCH1 gene were associated with reduced severity of persistent leg pain among 168 Caucasians with chronic, persistent lumbar root pain who underwent diskectomy. In this study, individuals were genotyped for 15 SNPs in the GCH1 gene. One year after surgery, 147 subjects completed the followup questionnaire, and among them, 5 SNPs in GCH1 were significantly associated with increased scores of persistent leg pain, which was the pre-specified primary outcome. Hence, GCH1 was shown to have a single haplotype. A single haplotype or haplotype block is a sequence of contigious SNPs in the DNA that are statistically associated [56]. The researchers also evaluated experimental pain sensitivity in two separate cohorts of healthy volunteers. Those homozygous for this haplotype exhibited reduced pain sensitivity to experimental pain compared to controls.

Additionally, Tegeder et al. [51] showed a haplotype block is associated with pain protection and, in normal volunteers, lower ratings of experimental pain stimuli. The aim of the study was to provide further evidence for pain protective effect of the GCH1 haplotype. This study consisted of 11 homozygous carriers of the GCH1 haplotype who were previously identified in an earlier study and 23 noncarrier controls who were matched by age and gender. The study was double blind in regard to participants' GCH1 genotype. The healthy volunteers were rated for sensitivity to pain from injury to the skin or capsaicin-evoked primary hyperalgesia. This verificatory study had small, but acceptable, sample sizes and revealed that compared to controls, carriers GCH1 upregulation was lower in carriers of GCH1 haplotype than in noncarriers. Subsequently, Lötsch et al. [57] reexamined the pain haplotype of 15 DNA positions of the GCH1 gene [52] and concluded that there was 100% sensitivity and specificity by screening for just 3 GCH1 gene variants instead of the originally stated 15. The 3 GCH1 SNPs that span the entire DNA range of the haplotype are rs 8007267 G > A in the 5′-untranslated region, rs3783641A > T in intron 1, and rs10483639C > G in the 3′ untranslated region [57]. 

Recently, Campbell et al. [50] also concluded that the GCH1 polymorphisms were associated with lower pain ratings in a group of healthy human volunteers who received induced painful stimuli. In this study of 39 healthy volunteers who participated in a neuroimaging PET study, individuals were genotyped and topical capsaicin cream was applied to the dorsal aspect of hand. Pain ratings were collected over a 90 minute interval. By using analysis of covariance, the researchers correlated the relationships between 5 SNPs and mean rating of experimental capsaicin pain. The individuals with the uncommon alleles reported 44% less pain than noncarriers.

In verificatory studies, Doehring and colleagues [55] conducted an observational cross-sectional analysis among Caucasian volunteers and concurred that the GCH1 pain protective effect is associated with the GCH1 haplotype. The study involved 523 patients who were enrolled in three different outpatient pain centers and had therapy for less than a month. Data were analyzed on 424 of 519 self-reported Caucasians participants. Both genotype and pain phenotype data were collected. The results indicate that lower levels of GCH1 expression have a pain protective effect. This decreased GCH1 upregulation may be prophylactic and delay the need for pain therapy. Patients with this haplotype needed shorter therapy than noncarriers [55]. 

In other studies, Lötsch et al. examined 251 unrelated individuals with cancer and pain [58]. The study subjects were homozygous carriers of the GCH1 variant, and it was noted that the time between cancer diagnosis and the need for opioid therapy initiation was longer than in heterogenous individuals. Hence, suggesting reduced GCH1 upregulation delays the need for opioid initiation in cancer treatment.

Two studies, however, failed to corroborate the GCH1 pain protective haplotype and decreased pain ratings. Lazarev et al. [59] examined genetic variations in the GCH1 gene in two SNPs (rs8007267 G > A and rs 3783641 A > T) and concluded that even with a large sample size of patients (236 Caucasians), significant pain patterns based on the GCH1 genotype could not be distinguished. The researchers concluded that the visceral pain pathway of pancreatitis may be different from that of neuropathic pain [59]. In another study, Kim and Dionne [60] failed to replicate the GCH1 gene variant is linked to lower pain response. This study examined healthy individuals with surgical removal of impacted molars. Their inconsistencies, however, may be related to their mixed ethnic sample, which induced population stratification and made it difficult to identify the pain phenotype [60].

## 3. Implications for Future Research and Practice

More work is needed to clearly define chronic pain phenotypes among individuals with SCD. Differences among varying phenotypic presentations also need to be studied. This need for more clinical research regarding SCD, chronic pain, and healthcare provision [37, 38] will help with improving the understanding of the chronic pain phenomenon, offer culturally appropriate interventions, and improve care for those who are vulnerable to disparate care. Research is needed to more clearly define chronic pain for those with SCD and guide the development of culturally and ethnically appropriate interventions to minimize complications and improve quality of life. There is the need and promise to personalize care based on individual genetic predisposition.

Research studies pertaining to chronic SCD pain should include genomics as a variable in a multidisciplinary approach. These studies should be conducted to identify relationships between pain reports and genomic markers, using appropriate designs such as candidate gene association studies. By using the candidate gene approach, researchers can have a focused view of genomic regions of interest. Hence, instead of fishing widely and possibly blindly in the human genome, correlation procedures may be applied to specifically identified SNPs in candidate genes associated with pain. By using translational research designs, bench findings may have clinical applicability in identifying factors that affect the heterogeneity of pain.

The challenges of prior research with this population, such as small sample sizes and lack of power, might be minimized with a multisite approach. In addition to quantitative inquiry, qualitative inquiry will capture subjective perspective, and advance our knowledge of this complex and debilitating condition. The blending of genomic data with chronic pain phenomena could enrich our interventions for people with SCD and chronic pain.

The functional impact of polymorphisms might identify one reason for variability or heterogeneity of pain phenotype in SCD. By using a holistic approach inclusive of genomics, researchers may better identify underlying mechanisms of chronic pain to ultimately influence clinical treatment decisions. Additionally, personalized healthcare will improve when we better understand the genetic component to pain and how this affects symptoms.

## 4. Conclusion

SCD is a complex and challenging condition accompanied by serious problems with chronic pain management. Pain protective genes have been implicated in chronic pain syndromes and could be applied to further study SCD. By using a candidate gene approach, researchers can evaluate interactions among many SNPs and the risk for SCD complications such as chronic pain. For example, analyzing individuals with SCD for mutations in GCH1 may help to explain the variability in pain ratings. Nurse scientists in a multidisciplinary team can contribute by developing innovative models that use multiple factors to predict individual's risks for disease development, symptom manifestation, and response to interventions. These important contributions can assist in early treatment of chronic pain and minimize sequela complications. 
